# Upregulation of KCNQ1OT1 promotes resistance to stereotactic body radiotherapy in lung adenocarcinoma by inducing ATG5/ATG12-mediated autophagy via miR-372-3p

**DOI:** 10.1038/s41419-020-03083-8

**Published:** 2020-10-20

**Authors:** Huanyu He, Xinmao Song, Zuozhang Yang, Yuchi Mao, Kunming Zhang, Yuanyuan Wang, Bin Su, Qiutian Li, Hong Chen, Yi Li

**Affiliations:** 1 Department of Oncology, 920th Hospital of Joint Logistics Support Force, 650032 Kunming, China; 2 Department of Radiation Oncology, Eye, Ear, Nose & Throat Hospital of Fudan University, 200031 Shanghai, China; 3 Department of Orthopaedics, The Third Affiliated Hospital of Kunming Medical University, 650118 Kunming, China; 4 Department of Pathology, 920th Hospital of Joint Logistics Support Force, 650032 Kunming, China

**Keywords:** Macroautophagy, Non-small-cell lung cancer

## Abstract

Stereotactic body radiotherapy (SBRT) has emerged as a standard treatment for non-small-cell lung cancer. However, its therapeutic advantages are limited with the development of SBRT resistance. The SBRT-resistant cell lines (A549/IR and H1975/IR) were established after exposure with hypofractionated irradiation. The differential lncRNAs were screened by microarray assay, then the expression was detected in LUAD tumor tissues and cell lines by qPCR. The influence on radiation response was assessed via in vitro and in vivo assays, and autophagy levels were evaluated by western blot and transmission electron microscopy. Bioinformatics prediction and rescue experiments were used to identify the pathways underlying SBRT resistance. High expression of KCNQ1OT1 was identified in LUAD SBRT-resistant cells and tissues, positively associated with a large tumor, advanced clinical stage, and a lower response rate to concurrent therapy. KCNQ1OT1 depletion significantly resensitized A549/IR and H1975/IR cells to radiation by inhibiting autophagy, which could be attenuated by miR-372-3p knockdown. Furthermore, autophagy-related 5 (ATG5) and autophagy-related 12 (ATG12) were confirmed as direct targets of miR-372-3p. Restoration of either ATG5 or ATG12 abrogated miR-372-3p-mediated autophagy inhibition and radiosensitivity. Our data describe that KCNQ1OT1 is responsible for SBRT resistance in LUAD through induction of ATG5- and ATG12-dependent autophagy via sponging miR-372-3p, which would be a potential strategy to enhance the antitumor effects of radiotherapy in LUAD.

## Introduction

Lung cancer is the second leading cause of cancer mortality worldwide^1^. More than 87% of patients have non-small-cell lung cancer, of which lung adenocarcinoma (LUAD) is the most predominant histological subtype, accounting for ~50% of patients^2^. Radiotherapy is the first-line treatment for this disease. Recently, due to the inherent advantages of stereotactic body radiation therapy (SBRT), including higher doses in a few fractions and more favorable toxicity than conventionally fractionated radiotherapy, SBRT to the lung has been developed and expanded. To date, SBRT is recommended as the standard of care in medically inoperable patients with lung cancer^3^. Unfortunately, the 3-year cumulative incidence of local failure, either alone or simultaneous with distant failure, still ranged from 4 to 19% after SBRT treatment^4^, suggesting a group of patients within LUAD may be more resistant to SBRT. Thus, exploring underlying mechanisms of SBRT resistance to reveal the related biomarkers is critical to optimize the clinical use of SBRT for lung cancer.

A class of non-protein coding transcripts longer than 200 nucleotides are defined as long noncoding RNAs (lncRNAs)^5^. LncRNAs emerge as a central player controlling diverse biologic processes via regulating a wide range of proteins at the transcriptional, post-transcriptional, and translational levels^6,7^. Most of the well-studied lncRNAs have been recently found to be crucial in the therapeutic resistance of cancer^8^. For instance, high levels of lncARSR correlated with inadequate response to sunitinib therapy in renal cancer, and bioactive lncARSR could disseminate sunitinib resistance via miR-34/miR-449 to facilitate AXL and c-MET expression^9^. Similarly, KCNQ1OT1 (Potassium voltage-gated channel subfamily Q member 1 opposite strand 1), one imprinted antisense lncRNA located in KCNQ1 loci on human chromosome 11p15.5^10^, has been reported to play an oncogenic role in several human cancers. Growing evidence showed that KCNQ1OT1 could influence the chemotherapeutic response via cell cycle arrest, apoptosis, and autophagy process^11,12^. However, the profile of lncRNAs involved with SBRT resistance has not been elucidated in LUAD.

Autophagy is an evolutionally conserved process to maintain cellular homeostasis by recycling proteins and organelles and targeting them for degradation^13^. Growing evidence identified that autophagy could help cancer cells survive under acute stress, such as in response to therapy, thereby favoring therapeutic resistance^14^. The molecular control of autophagy is an elaborate multistep process, mainly regulated by conserved autophagy-related genes (ATG genes), which can form several functional complexes associated with other regulators involved in autophagy initiation and execution. For example, the ATG5-ATG12-ATG6L1 complex acts as an E3-like enzyme to catalyze the conjugation of LC3B-I with phosphatidylethanolamine (PE) to produce LC3B-II. The conversion from LC3B-I to LC3B-II is essential in autophagosome formation, and LC3B-II represents a biomarker for autophagy^15^. Previous studies have shown that autophagy inhibition, such as targeting specific ATG genes, could be an effective strategy to enhance the antitumor response while reducing toxicity in cancers^16,17^. In the context, it is urgent to deeply understand whether and how SBRT-resistant cells utilize autophagy to avoid killing effects of IR, which could reveal potential targets for selective and specific inhibition.

In this study, we described that KCNQ1OT1 is upregulated in IR-resistant LUAD cells and tumor tissues, which is associated with a low objective response rate and poor prognosis in LUAD patients. We further investigated a novel role of KCNQ1OT1 in radiotherapy response in vitro and in vivo and showed KCNQ1OT1 depletion resensitized IR-resistance cells to irradiation via inhibiting autophagy. Mechanistically, we explored the involvement of miR-372-3p and its autophagy-related targets (ATG5 and ATG12) in KCNQ1OT1 depletion-induced radiosensitization, presenting a potential therapeutic strategy to overcome SBRT resistance in LUAD.

## Results

### KCNQ1OT1 is upregulated in IR-resistant cells and associated with the poor response of anticancer treatment of LUAD patients

**Fig. 1:**
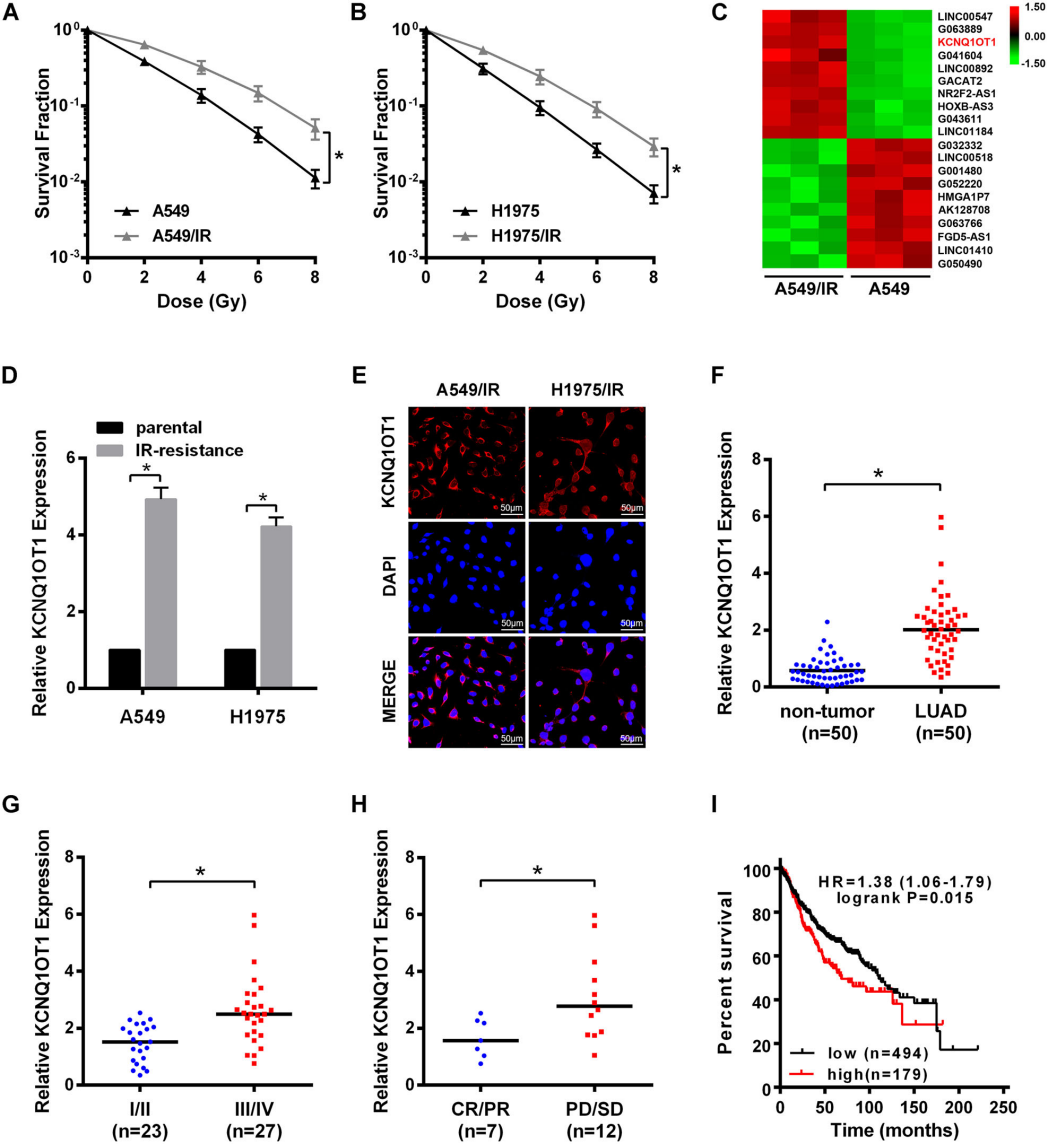
KCNQ1OT1 was upregulated in IR-resistant cells and associated with the poor response of anticancer treatment of LUAD patients. **A**, **B** Colony survival assay in parental and IR-resistant A549 (**A**) and H1975 (**B**) cells exposed with indicated irradiation dosage. **C** LncRNAs microassay data of parental and IR-resistant A549 cells were shown in the Heatmap. **D** qRT-PCR analysis of KCNQ1OT1 expression in parental and IR-resistant A549 and H1975 cells. GAPDH was used as an internal control. **E** Representative images of FISH analysis of KCNQ1OT1 expression in IR-resistant A549 and H1975 cell, Scale bars represent 50 μm. **F–H** qRT-PCR analysis of KCNQ1OT1 expression in matched tumor and non-tumor LUAD specimens (*n* = 50, **F**), in LUAD tumor tissues with different TNM stages (*n* = 50, **G**), and in LUAD tissues from patients with completed or partial response (CR/PR, *n* = 7) and patients with stable or progressive disease (SD/PR, *n* = 12) after concurrent chemoradiotherapy. **H** The expression levels were presented as the fold change normalized to GAPDH and relative to one non-tumor tissue. **I** Overall survival for 673 LUAD patients with high level or low-level tumor expression of KCNQ1OT1 via Kaplan–Meier Plotter (http://kmplot.com/analysis/). Data are presented as the mean ± SD. from three independent experiments, *p < 0.05.

To mimic the residual cells after SBRT treatment, the IR-resistant cell lines (A549/IR and H1975/IR) were established after exposing A549 and H1975 cells to 18 Gy irradiation in three fractions, respectively. As shown by colony survival assay, A549/IR and H1975/IR cells exhibited higher growth ability and increased survival fractions after IR treatment, and the SER10 value was decreased compared with their individual parental cells (SER10: 0.76 vs. 1; and 0.79 vs.1, Fig. 1A, B). Next, the profiles of lncRNAs required for SBRT resistance were screened between A549/IR and A549 cells by microarray analysis (Fig. 1C). From the top 10 upregulated lncRNAs, we validated the endogenous level of KCNQ1OT1 was the most upregulated in both A549/IR and H1975/IR cells compared to their parental cells by qRT-PCR (Fig. 1D, Fig. S1), and KCNQ1OT1 was predominantly expressed in the cytoplasm by FISH assay (Fig. 1E). Moreover, KCNQ1OT1 expression was measured in 50 LUAD samples via qRT-PCR. As compared with paired non-tumor tissues, KCNQ1OT1 was significantly increased in pretherapy LUAD tissues (Fig. 1F) with a mean value of 2.11, which was used to divide LUAD samples into high and low KCNQ1OT1 groups. Additionally, the higher level of tumor KCNQ1OT1 was observed in stage III and IV patients than that of stage I and II patients (Fig. 1G). Interestingly, among the 19 patients receiving concurrent chemoradiotherapy, we found high KCNQ1OT1 expression trended towards poor therapeutic response. KCNQ1OT1 expression was significantly increased in patients with stable or progressive disease (*n* = 12) compared to those with completed or partial response (*n* = 7) (Fig. 1H), and objective response rate (ORR) was relatively lowered in the high KCNQ1OT1 group versus the low KCNQ1OT1 (3/11 vs. 4/8). Furthermore, high KCNQ1OT1 expression was confirmed to correlate with large tumor size and an advanced clinical stage (Table 1). Finally, Kaplan–Meier Plotter analysis was performed in 673 LUAD patients from TCGA database, shown that LUAD patients with high KCNQ1OT1 level experienced a shorter overall survival (OS) than those with low KCNQ1OT1 level (Fig. 1I)

**Table 1:** Correlations between KCNQ1OT1 and clinicopathologic features of LUAD patients.

	Total	No. of patients^a^		Chi-square test *P* value
		High KCNQ1OT1 (24)	Low KCNQ1OT1 (26)	
Gender				0.412
Male	28	12	16	
Female	22	12	10	
Age				0.094
≤60	21	13	8	
>60	29	11	18	
Tumor size				0.011*
≤5 cm	24	7	17	
>5 cm	26	17	9	
Location of tumor				0.981
Left (upper/lower)	23	11 (3/8)	12 (2/10)	
Right(upper/middle/lower)	27	13 (2/5/6)	14 (2/6/6)	
Differentiation				0.089
Well	10	5	5	
Moderately	22	7	15	
Poorly	18	12	6	
Clinical stage				0.021*
I/II	23	7	16	
III/IV	27	17	10	

*P < 0.05.

^a^The mean expression of KCNQ1OT1 in lung adenocarcinoma tissues was used as a cutoff to divide samples into a high or low subgroup.

### KCNQ1OT1 knockdown represses IR resistance of LUAD in vitro and in vivo

**Fig. 2:**
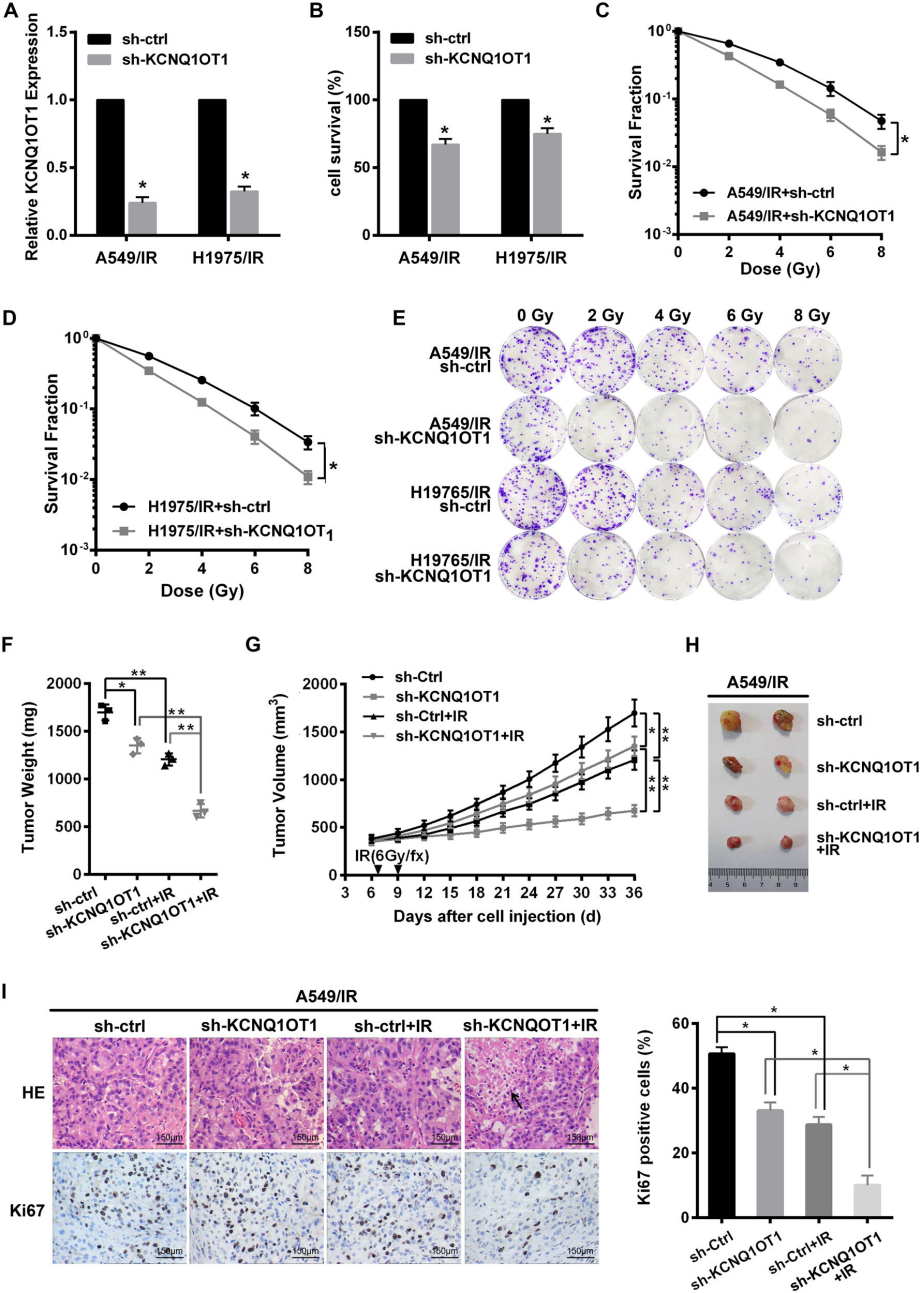
KCNQ1OT1 knockdown repressed IR resistance of LUAD in vitro and in vivo. **A** qRT-PCR analysis of KCNQ1OT1 expression in A549/IR and H1975/IR cells after infected with a control (sh-ctrl) or KCNQ1OT1 shRNA knockdown (sh-KCNQ1OT1) lentiviral vector. **B** Cell viability of KCNQ1OT1 knockdown and control A549/IR and H1975/IR via CKK8 assay. **C**–**E** Colony survival assay in KCNQ1OT1 knockdown and control A549/IR (**C**) and H1975/IR (**D**) cells exposed with indicated irradiation dosage. Representative images were presented (**E**). **F**–**H** The weights (**F**) and volumes (**H**) of subcutaneous xenografts from KCNQ1OT1 knockdown and control A549/IR cells in nude mice with or without IR, *n* = 3 in each group. Representative images of the xenografts were presented (**H**). **G** H&E staining, Ki67 expression by IHC in the xenografts derived from KCNQ1OT1 knockdown and control A549/IR cells with or without irradiation. Scale bars: 150 μm. The black arrow represents necrosis. The histograms showed the average Ki67 proliferative index of each group. Data are presented as the mean ± SD. from three independent experiments, *p < 0.05.

To evaluate whether KCNQ1OT1 affected radiotherapy response in LUAD cells, the expression of KCNQ1OT1 in A549/IR and H1975/IR cells were stably knocked down by lentivirus-mediated shRNA (sh-KCNQ1OT1), and their control cell lines were generated via an empty vector control lentivirus (sh-ctrl) (Fig. 2A). We found that KCNQ1OT1 depletion markedly suppressed the proliferation of IR-resistant cells via CKK8 assay (Fig. 2B). Meanwhile, as shown by colony survival assay, KCNQ1OT1 depletion sensitized A549/IR and H1975/IR cells to radiation, with fewer and smaller colony formation and higher SER value than their control cells (SER10: 1.25 vs. 1; and 1.19 vs. 1, Fig. 2C–E). Furthermore, the above effect of KCNQ1OT1 depletion was validated using an in vivo mouse model. A549/IR cells with sh-KCNQ1OT1 or control shRNA were injected subcutaneously into nude mice. Seven days later, the mice bearing xenografts were treated with or without irradiation, then the tumor volumes were measured every three days. We found that xenografts from sh-KCNQ1OT1 A549/IR cells grew slower compared to control tumors in terms of tumor weight and tumor volume. And the growth of control tumors was significantly inhibited by IR treatment. Importantly, KCNQ1OT1-depleted tumors were significantly more sensitive to IR, with a dramatic tumor growth delay when compared with KCNQ1OT1 depletion alone or the control with IR (Fig. 2F–H). And a lower Ki67 proliferation index using immunostaining assay was observed in combination with KCNQ1OT1 depletion and IR, as well as more necrosis compared with the control group (Fig. 2I).

### KCNQ1OT1 knockdown inhibits autophagy in IR-resistant LUAD cells

**Fig. 3:**
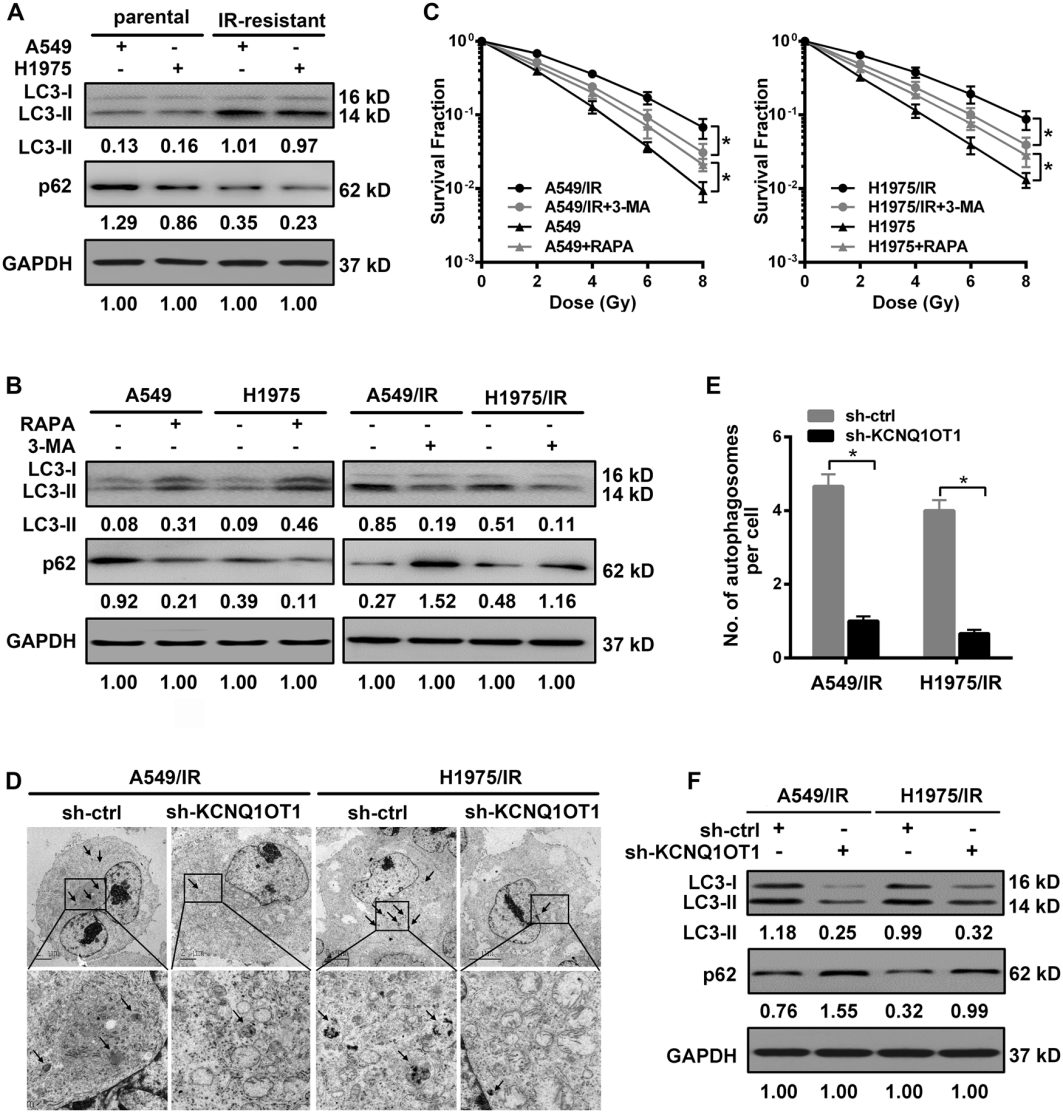
KCNQ1OT1 knockdown inhibited autophagy in IR-resistant LUAD cells. **A** Western Blot analysis for autophagy-relative protein LC3B-II and p62 in parental and IR-resistant A549 and H1975 cells with GAPDH as an endogenous control. **B** The expression of LC3B-II and p62 protein in cells after treatment with or without 2 mM 3-MA or 100 nM RAPA for 12 h with GAPDH as an endogenous control. **C** Colony survival assay in cells treated with or without 2 mM 3-MA or 100 nM RAPA for 12 h and followed by indicated radiation dosages. **D** Transmission electron microscopy (TEM) to observe the ultrastructure features in control and KCNQ1OT1-knockdown A549/IR and H1975/IR cells. Scale bars: 2 μm for A549/IR cells, 5 μm for H1975/IR cells. Black arrows represent autophagosomes with double membranes. **E** The average number of autophagosomes per cell was calculated statistically in A549/IR and H1975/IR cells. **F** Western Blot analysis for LC3B-II and p62 protein in control and KCNQ1OT1-knockdown A549/IR and H1975/IR cells with GAPDH as an endogenous control. Data are presented as the mean ± SD. from three independent experiments, *p < 0.05.

Autophagy has been implicated in therapeutic resistance^18^. We thus evaluated the autophagy activity during IR resistance in LUAD. Here, autophagy was quantified by western blot assay with both cleavages of LC3-I protein to LC3-II, as an indicator of complete autophagosomes, and expression of autophagy substrate p62 protein. We found autophagy was markedly activated in IR-resistant cells, with an increasing level of LC3-II (normalized to GAPDH) and a decreased p62 level compared to their parental cells (Fig. 3A). Then, to further evaluate the effect of autophagy and IR response in LUAD, the IR-resistant cells and their parental cells were subjected to 3-MA as an autophagy inhibitor and RAPA to induce autophagy, respectively. As shown in Figs. 3B, 3-MA markedly reduced LC3-II level along with increased p62 expression in both A549/IR and H1975/IR cells, while RAPA treatment led to an opposite trend in their parental cells. After confirmation of the above effect on autophagy, cells were exposed to various doses of IR. Colony survival assay showed that 3-MA treatment significantly sensitized both IR-resistant cells to irradiation (Fig. 3C). In contrast, a higher capacity of colony formation was observed upon PARA intervention (Fig. 3C). Given the above results, we, therefore, tested whether KCNQ1OT1 regulates autophagy in LUAD cells. As shown by transmission electron microscopy (TEM), the number of autophagosomes, characterized as sequestration of cytoplasmic constituents with double membranes^19^, was decreased in both KCNQ1OT1-depleted A549/IR and H1975/IR cells compared to their controls (Fig. 3D, E). Consistently, western blot assay also showed autophagy activation in both IR-resistant cells was dramatically abolished by KCNQ1OT1 depletion. As compared with their controls, a visible suppression of lapidated LC3 level (LC3-II) along with an increase of P62 protein was observed in KCNQ1OT1-depleted A549/IR and H1975/IR cells (Fig. 3F).

### miR-372-3p bound by KCNQ1OT1 confers radiosensitivity in LUAD cells via autophagy inhibition

**Fig. 4:**
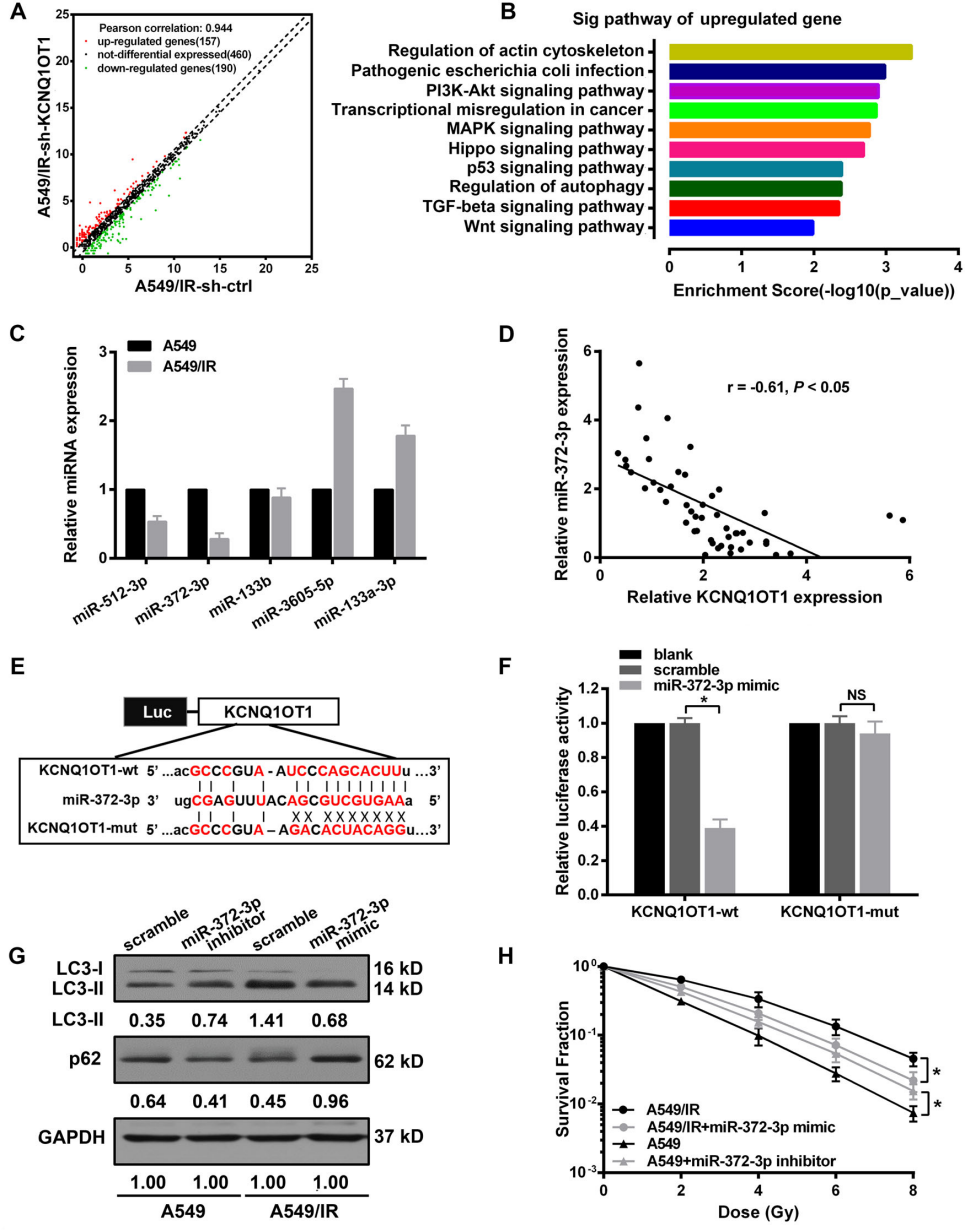
miR-372-3p bound by KCNQ1OT1 conferred radiosensitivity in LUAD cells via autophagy inhibition. **A** Scatter plot of differentially expressed miRNAs between KCNQ1OT1 knockdown and control A549/IR cells. **B** KEGG (Kyoto Encyclopedia of Genes and Genomes) pathway analysis of upregulated miRNAs in KCNQ1OT1-knockdown A549/IR cells, and “regulation of autophagy” was included among the top 10 significant pathways. **C** qRT-PCR analysis for differentially expressed miRNAs in parental and IR-resistant A549 cells. **D** Regression analysis for the correlation between KCNQ1OT1 and miR-372-3p in LUAD tumor specimens, *n* = 50. **E** Schematic illustration of the potential binding sites of miR-372-3p (*middle*) and KCNQ1TO1 (KCNQ1OT1-wt, *top*), and the designed mutant sequence (KCNQ1OT1-mut, *bottom*). **F** Luciferase assay in HEK293 cells after cotransfected either KCNQ1OT1-wt or KCNQ1OT1-mut vectors with miR-372-3p mimic. **G** Western Blot analysis for LC3B-II and p62 protein in miR-372-3p inhibiting A549 and miR-372-3p-expressing A549/IR cells with GAPDH as an endogenous control. **H** Colony survival assay in miR-372-3p inhibiting A549 and miR-372-3p-expressing A549/IR cells with indicated irradiation dosages. Data are presented as the mean ± SD. from three independent experiments, *p < 0.05.

Due to the cytoplasmic expression of KCNQ1OT1, we hypothesized the molecular mechanism of KCNQ1OT1 on autophagy is attributed to the role of competitive endogenous RNA (ceRNA), which could sponge miRNA to regulate the downstream gene expression^20^. Therefore, a microarray assay was performed to screen the differentially expressed miRNAs associated with KCNQ1OT1 depletion. A total of 347 miRNAs (157 upregulated and 190 downregulated) were found in sh-KCNQ1OT1 A549/IR cells compared with controls (Fig. 4A). The top 10 significant signaling pathways were listed based on the Kyoto Encyclopedia of Genes and Genomes (KEGG) analysis with the upregulated panel, wherein autophagy was significantly influenced (Fig. 4B). Furthermore, the miRNAs containing complementary base pairs with KCNQ1OT1 were searched via the Starbase database, followed by overlapping the top 20 upregulated miRNAs, potential candidates including miR-512-3p, miR-372-3p, miR-133b, miR-3605-5p, and miR-133a-3p were identified. Then, the expression of individual miRNA was measured by qPCR in IR-resistant and parental cells. It was shown that miR-372-3p expression in A549/IR was the lowest among the five miRNAs compared to parental cells (Fig. 4C). We further confirmed the direct interactions between miR-372-3p and KCNQ1OT1, miR-372-3p mimic was cotransfected with either recombinant wide-type or mutant pmirGLO-KCNQ1OT1 vector in HEK293 cells. The luciferase activity of the KCNQ1OT1-wt vector was dramatically suppressed by miR-372-3p mimics, whereas the above effect was markedly abolished by a pmirGLO-KCNQ1OT1-mut vector (Fig. 4E, F). Additionally, we found that miR-372-3p was negatively correlated with KCNQ1OT1 expression in LUAD tumor samples (Fig. 4D). Moreover, the role of miR-372-3p in IR response was evaluated after transfection with either miR-372-3p mimic or inhibitor, respectively. We found miR-372-3p overexpression in A549/IR reduced the capacity of colony formation, with a 17.6% increase of SER value than scramble A549/IR cells. In contrast, miR-372-3p knockdown increased resistance to IR, and the SER value was reduced to 88.5% compared to scramble A549 cells (Fig. 4H). Additionally, western blot results showed autophagy was inhibited in miR-372-3p overexpressed A549/IR cells, with a markedly decreased LC3-II and elevated p62 level. Whereas, increased LC3-II and reduced p62 protein was observed in miR-372-3p depleted A549 cells compared to scrambled cells (Fig. 4G).

### miR-372-3p inhibition attenuates KCNQ1OT1 depletion-induced radiosensitivity through regulation of ATG5 and ATG12

**Fig. 5:**
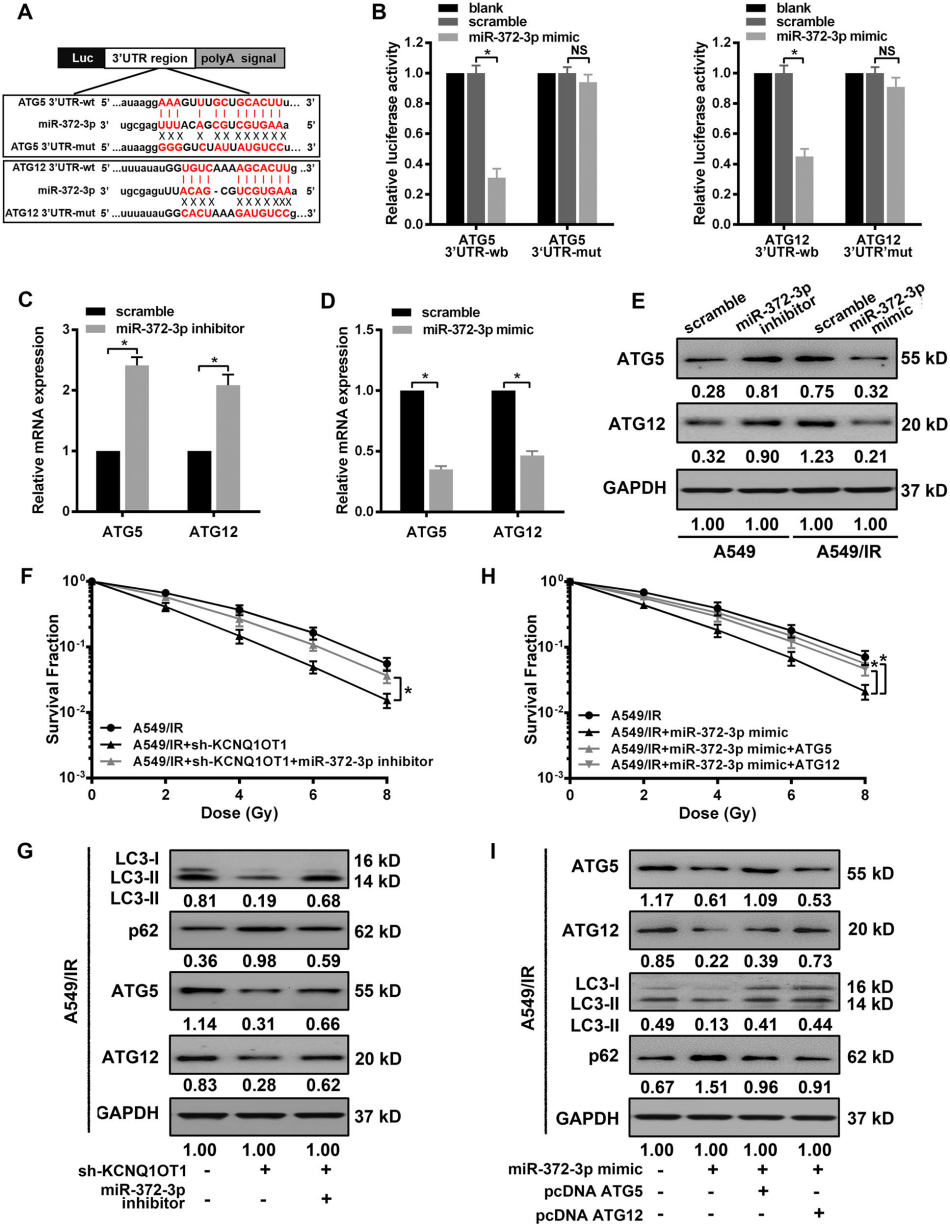
miR-372-3p inhibition attenuated KCNQ1OT1 depletion-induced radiosensitivity through regulation of ATG5 and ATG12. **A** Schematic illustration of the potential binding sites for miR-372-3p in the 3′-UTRs of ATG5 (upper) and ATG12 (lower). **B** Dual-luciferase assay in HEK293 cells after cotransfected either wild-type (ATG5-wt and ATG12-wt) or mutant (ATG5-mut and ATG12-mut) vectors with miR-372-3p mimic. **C**, **D** qRT-PCR assay for mRNA level of ATG5 and ATG12 in miR-372-3p inhibiting A549 (**C**) and miR-372-3p-expressing A549/IR (**D**) cells. **E** Western blot assay for protein level of ATG5 and ATG12 in miR-372-3p inhibiting A549 and miR-372-3p-expressing A549/IR cells with GAPDH as an endogenous control. **F** Western blot analysis for the expression of LC3-II, p62, ATG5, ATG12, and GAPDH (for normalization) in KCNQ1OT1-knockdown A549 IR cells with or without miR-372-3p inhibitor transfection. **G** Colony survival assay in KCNQ1OT1-knockdown A549 IR cells with or without miR-372-3p inhibitor transfection. **H** Western blot analysis for the expression of ATG5, ATG12, LC3-II, p62, and GAPDH (for normalization) in miR-372-3p-expressing, miR-372-3p/ATG5-expressing, or miR-372-3p/ATG12-expressing A549/IR cells. **I** Colony survival assay in miR-372-3p-expressing, miR-372-3p/ATG5-expressing, or miR-372-3p/ATG12-expressing A549/IR cells. Data are presented as the mean ± SD. from three independent experiments, *p < 0.05.

MiRNAs are functionally dependent on the downstream genes, whose 3′UTR owns partial or complete sequence homology to specific miRNA. Given the above effect of miR-372-3p on autophagy, we searched its potential targets relevant to autophagy via miRNA databases (PITA, miRmap, and miRanda). ATG5 and ATG12, two critical factors in autophagosome formation and elongation, were predicted candidates of miR-372-3p (Fig. 5A). The potential binding between their 3′-UTR and miR-372-3p were verified by dual-luciferase reporter assay. As expected, miR-372-3p mimic led to a decreased luciferase activity in the wild-type vector of ATG5 or ATG12, but the inhibited effects were abolished by mutant miR-372-3p-targeting sequences in their 3′-UTR (Fig. 5B). Additionally, we explored the regulating effect of miR-372-3p on the expression of ATG5 or ATG12. miR-372-3p knockdown in inhibitor-transfected A549 cells promoted both mRNA and protein expression of ATG5 and ATG12 compared to scrambled A549 cells (Fig. 5C, E). While MiR-372-3p overexpression in the presence of its mimic showed a significant decrease of ATG5 and ATG12 in mRNA and protein levels in A549/IR cells (Fig. 5D, E). Due to the above results, we hypothesized miR-372-3p-mediated ATG5 and ATG12 expression could be involved in KCNQ1OT1 depletion-induced radiosensitivity. Firstly, the miR-372-3p inhibitor was transfected into KCNQ1OT1-depleted A549/IR cells, and we found miR-327-3p knockdown significantly abolished KCNQ1OT1 depletion-induced inhibition on colony survival ability, with a 14.2% decrease in SER10 value compared with sh-KCNQ1OT1 A549/IR cells (SER10: 1.34 vs. 1.15). Moreover, miR-372-3p knockdown also attenuated autophagy inhibition mediated by KCNQ1OT1 depletion, which increased the protein level of LC3-II and decreased p62 level compared with sh-KCNQ1OT1 cells. Importantly, we observed the protein levels of ATG5 and ATG12 were markedly decreased in KCNQ1OT1-depleted A594/IR cells, whereas their trends were partially antagonized when cotransfected with miR-372-3p inhibitor. Next, to validate whether the effect of miR-372-3p on radiation response was dependent on ATG5 or ATG12, the expression vectors lacking 3′-UTR region were established, named as pcDNA-ATG5 or pcDNA-ATG12, which could escape the upstream regulation of miR-372-3p. The ectopic expressions of ATG5 or ATG12 were confirmed in A549/IR cells after cotransfected with the individual vector and miR-372-3p mimic, with higher protein levels than miR-372-3p-expressing cells. Meanwhile, we found restoration of either ATG5 or ATG12 antagonized the miR-372-3p-mediated sensitivity to radiation, which also alleviated the decrease of autophagy activity induced by miR-372-3p.

## Discussion

Recently, the use of SBRT for NSCLC has been clinically optimized by some tumor- and treatment-related factors to predict failures, such as larger tumor size and lower radiation doses^21,22^. However, Resistance to SBRT is still a significant burden in LUAD therapy. Whether groups with differentially expressed genes could be more likely to respond to SBRT is unclear. In this study, we generated monoclonal IR-resistant cell lines to mimic SBRT resistance in the clinic via hypofractionated radiation. We found that IR-resistant cells, which could maintain the resistant phenotype in the absence of IR over 30 passages (unpublished data), enhanced proliferation and radioresistance in comparison to their parental cell lines. Then, these models were used to explore the underlying mechanisms responsible for the above phenomenon. KCNQ1OT1 was screened out as one of the most upregulated lncRNAs via microarray assay. In accordance with previous reports in several human cancers^23,24^, a high level of KCNQ1OT1 was found in our LUAD cohort, which correlated with large tumor size, an advanced clinical stage and shorter survival of patients. Recent studies indicating the association with other malignant characteristics, such as poor differentiation^12^ and positive lymphatic metastasis^25^, further supported that KCNQ1OT1 served as a poor prognostic factor for LUAD. However, a controversial correlation between KCNQ1OT1 and prognosis has also been reported in lung cancer^26^. The possible reason may be due to the sample size, different cutoff value and other factors. In addition, we noted that LUAD patients with high KCNQ1OT1 expression had poor ORR after concurrent chemoradiotherapy, indicating that KCNQ1OT1 might be a potential predictor of therapeutic response. Moreover, our study revealed enhanced KCNQ1OT1 expression in IR-resistant cells (A549/IR and H1975), and their radiosensitivity was recovered in vitro and in vivo by KCNQ1OT1 knockdown. To our knowledge, the involvement of KCNQ1OT1 in radiation resistance has not been reported before. It is therefore expected that a KCNQ1OT1-targeted therapy could be a promising strategy against SBRT resistance in LUAD.

Accumulated evidence showed that increased autophagy is an adaptive response that contributes to the acquisition of radiation resistance in cancer^27^. Indeed, we observed IR-resistant cells with high expression of KCNQ1OT1 enhanced autophagy activity compared to their parental cells. Considering that KCNQ1OT1 as a regulator of autophagy was correlated with chemoresistance in some cancers like colorectal cancer and NSCLC^24,28,29^, we speculated elevated KCNQ1OT1 in IR-resistant cells might keep them from IR damage by the promotion of autophagy. In the present study, we revealed that KCNQ1OT1 depletion significantly reduced autophagy in IR-resistant cells, based on the protein expression of LC3-II and p62, as well as the lower number of autophagosomes via TME assay. It was reported that the repression of autophagy sensitized lung cancer cells to radiotherapy^30^. Following this finding, we found autophagy inhibitor 3-MA markedly restored the radiation response of IR-resistant cells, and this sensitive effect was consistently observed in KCNQ1OT1 depletion. In contrast, treatment with RAPA led to a significantly increased resistance towards IR in the parental cells. Taken together, the above results suggest that KCNQ1OT1-mediated autophagy exerts a crucial function in the SBRT resistance of LUAD.

What molecular mechanism, exactly, is responsible for the KCNQ1OT1-mediated autophagy in IR response? Recent studies reported that lncRNAs located in the cytoplasm always function as ceRNAs, which could regulate miRNA-mRNA axis via competitively binding with miRNAs. For example, lncRNA LCAT1 promoted tumorigenesis and metastasis of lung cancer via sponging miR-4715-5p to upregulate the RAC1 activity^31^. In our study, we detected downstream miRNAs expression after sh-KCNQ1OT1 transfection by microarray and found that miR-372-3p was the most upregulated. Then this inversely expressed the relationship between KCNQ1OT1 and miR-372-3p was confirmed in LUAD tissue and IR-resistance cells. Their direct binding was further verified via luciferase reporter assay. Moreover, overexpression of miR-372-3p inhibited autophagy and enhanced radiosensitivity of IR-resistant cells. Conversely, the introduction of miR-372-3p inhibitor abolished the inhibition of autophagy and the sensitive effect mediated by KCNQ1OT1 depletion. The above findings reveal that miR-327-3p is a possible ceRNAs of KCNQ1OT1, which is involved in KCNQ1OT1-mediated autophagy in IR resistance. Although KCNQ1OT1 has been reported to act as a regulator of miRNAs in several cancers previously^23,32–35^, this study describes a new downstream target miR-372-3p in KCNQ1OT1 regulation, which may be dependent on the variety of cell and tissue context.

**Fig. 6:**
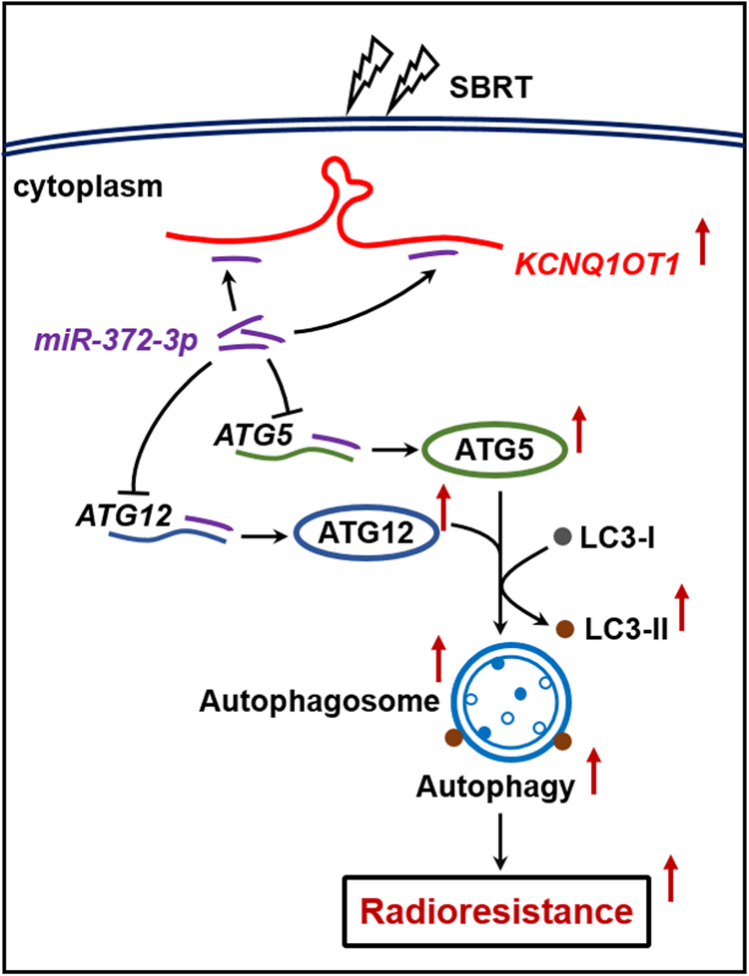
The Schematic cartoon of the mechanism of KCNQ1OT1-induced SBRT resistance in LUAD through the promotion of ATG5- and ATG12-dependent autophagy via sponging miR-372-3p. The red arrows represent promotion; The black arrows with flat end represent inhibition.

MiRNAs have been reported to regulate autophagy via binding the 3′UTR of downstream target genes in human cancers^36^. For example, miR-214 inhibited IR-induced autophagy via targeting ATG12 to promote radiosensitivity^37^. MiR-142-3p reduced sorafenib-induced autophagy through the downregulation of ATG5/ATG16L1 in hepatocellular carcinoma cells^38^. In this study, bioinformatics analysis predicted that miR-372-3p might target the 3′-UTR of ATG5 and ATG12, and we further verified their direct interaction and the inverse regulatory role of miR-372-3p in both mRNA and protein expressions of these proteins. Both ATG5 and ATG12 are thought to be dispensable in the developing autophagosome. ATG12 is located upstream of the LC3B system, which is finally conjugated to ATG5 to form the irreversible ATG12-ATG5 complex, then contributes to the elongation of autophagosomal membranes. Given that ectopic expression of either ATG5 or ATG12 could activate autophagy in vitro and in vivo^39,40^, we tested whether ATG5 and ATG12 is involved in radiation response regulated by KCNQ1OT1/miR-372-3p axis. In this study, a reduced level of ATG5 and ATG12 protein was observed in KCNQ1OT1-depleted IR-resistance cells. Importantly, restoration of either ATG5 or ATG12 cDNA rescued the above effects of miR-372-3p in LUAD cells, which led to an increase of autophagy and more resistance to IR treatment. These data reveal that KCNQ1OT1 sponging miR-372-3p promotes ATG5- and ATG12-dependent autophagy, thus leading to SBRT resistance in LUAD cells, as schematically presented in Fig. 6.

Several issues of current studies will require further exploration. First, owing to the complexity of the regulatory network in the organism, other molecules were not excluded as possibly being involved in KCNQ1OT1-induced autophagy activation. Thus, the identification of more target genes could help us better understand the network of SBRT resistance. Second, posttranslational modifications (PTMs), such as phosphorylation, ubiquitination, acetylation, and proteolysis, play a crucial role in modulating the function of the ATG proteins. In particular, their ability to interact with macroautophagy regulators^41^. Elucidation of the role of KCNQ1OT1 in PTMs of ATG5 and ATG12 will provide new connections between autophagy and KCNQ1OT1-induced radioresistance.

In conclusion, our results demonstrate that KCNQ1OT1 upregulation is a frequent event in LUAD, especially in patients with less response to anticancer treatment. Additionally, for the first time, our data reveal KCNQ1OT1 knockdown in SBRT-resistant cells significantly enhanced radiosensitivity both in vitro and in vivo by autophagy inhibition. Furthermore, we showed that KCNQ1OT1-induced SBRT resistance is attributed to sponging miR-372-3p through directly promoting ATG5- and ATG12-dependent autophagy in LUAD cells. Thus, the KCNQ1OT1/miR-372-3p axis via targeting ATG5 and ATG12 provides a new avenue to understand the mechanism of SBRT resistance in LUAD, which may have significant implication in LUAD intervention.

## Methods and materials

### Tissue specimens and survival analysis

A total of 50 patients who suffered lung adenocarcinoma were collected after biopsy or surgical resection between January 2016 and December 2018 at 920th Hospital (Yunnan, China). The adjacent healthy tissues were obtained 2 cm away from the tumor edge. Two independent oncologists estimated the staging of the disease according to the 8th edition of the TNM classification for lung cancer^42^. No patients received any anticancer therapy before sampling. Nineteen Patients without operative indications were treated with concurrent chemoradiotherapy. The therapeutic response was assessed by RECIST V.1.1^43^. All specimens were frozen in liquid nitrogen immediately for further analysis. The Institutional Ethics Committee at 920th hospital approved this research, and informed consent based on the Declaration of Helsinki was obtained from all patients. The correlation between KCNQ1OT1 expression and overall survival of LUAD patients were analyzed online by Kaplan–Meier Plotter (http://kmplot.com/analysis/).

### Cell culture and irradiation treatment

Human embryonic kidney cell line HEK293 and human lung adenocarcinoma cell lines A549 and H1975 were purchased from American Type Culture Collections (ATCC, Manassas, USA). Cells were maintained in RPMI-1640 medium (Gibco USA) with 10% fetal bovine serum (Sigma, USA), 500 units/L penicillin, and 500 μg/L streptomycin at standard conditions (37 °C, 5% CO_2_). Autophagy intervention was performed using either 2 mM 3-methyladenine (3-MA; Sigma, USA) or 100 nM Rapamycin (RAPA; Sigma, USA) for 12 h. External beam radiation was delivered on a 300KV X-ray machine (HITACHI, Japan) at room temperature. To generate radiation resistant cells, a total dose of 18 Gy in three fractions was delivered to parental A549 and H1975 cells over the course of 7 days. According to the method reported previously^44^, monoclonal cell lines with the highest radioresistance was obtained and designated as A549/IR and H1975/IR, respectively.

### Cell infection and transfection

KCNQ1OT1 shRNA and negative control shRNA were synthesized by Genechem (Shanghai, China). The target sequences were listed as followed: sh-KCNQ1OT1: 5′-TTGCTGGTTACTGGCTTGAAA-3′; sh-ctrl: 5′-TTCTCCGAACGTGTCACGT-3′. After packaging in HEK293 cells, recombinant lentiviruses were infected into cells and incubated for 4 h. Then, complete medium was added into the infected cells after discarding the supernatant. Mature hsa-miR-372-3p mimic, has-miR-372-3p inhibitor, and individual scramble (negative control) were purchased from RuiBoBio (Guangzhou, China). The CDS sequence of AGT5 or ATG12 without the 3′UTR was cloned into the pcDNA-3.0 plasmid to construct the pcDNA-ATG5 or pcDNA-ATG12 vector. Transfection was carried out with Lipofectamine 3000 Kit (Invitrogen, USA) and Opti-MEM serum-free medium (Invitrogen, USA) according to the manufacturer’s instructions.

### Cell proliferation assay

2 × 10^3^ Cells per well were plated into 96-well plates, and 10 μL of CKK8 reagent (Cell Counting Kit-8, Beyotime, China) was added into each well 96 h later. Then the optical absorbance value of each well was measured at 450 nm after incubation for 1 h.

### Colony formation assay

Cells were plated into six-well plates with different numbers and were irradiated with a single dose of 0, 2, 4, 6, or 8 Gy. Fourteen days later, cells were stained with crystal violet, and colonies more than 50 cells were counted. Survival Fraction (SF) was calculated as: SF = no. of colonies formed after individual dose / (no. of cells seeded × PE), where PE of the corresponding group was used as normalization and defined by: PE = no. of colonies formed for nonirradiation/no. of cells seeded. The value of the sensitization enhancement ratio (SER10) was calculated as previously described^45^.

### Quantitative real-time PCR

Total RNA was isolated from tissue samples and cell lines via TRIZOL reagent (Invitrogen, USA) and was synthesized into complementary (cDNA) via the Reverse Transcription Kit with specific RT-primers (Takara, Japan). Quantitative PCR was performed on the ABI Prism 7900 (Applied Biosystems, USA) using the SYBR PCR master mix (Takara, Japan) with specific PCR primers, listed in Table S1. The relative expression of individual genes was analyzed by the 2^-△△Ct^ method^46^. Glyceraldehyde 3-phosphate dehydrogenase (GAPDH) was used as the internal control for KCNQ1TO1 and mRNA expression, and U6 for miRNAs expression.

### Western blot assay

Protein was extracted from cells by a protein extraction reagent (Roche, Switzerland), and measured by a BCA protein kit (Pierce Biotechnology, USA). Forty microgram protein for each sample was electrophoresed in 10–15% SDS gel, then transferred to nitrocellulose membranes. After blocked with 5% fat-free milk at room temperature for 1 h, membranes were incubated with primary antibodies at a dilution of 1/1000 at 4 °C overnight, including anti-LC3 (#3868, Cell Signal Technology), anti-p62 (ab91526, Abcam), anti-ATG5 (#12994, Cell Signal Technology), anti-ATG12 (sc-271688, Santa Cruz), and anti-GAPDH (ab8245, Abcam). Then, followed with a secondary antibody (1:5000) at 37 °C for 1 h. The signals were detected by ECL Kit (Pierce Biotechnology, USA), and were analyzed by Image pro plus software.

### Transmission electron microscopy (TME)

Cells were fixed with a solution containing 3% glutaraldehyde plus 2% paraformaldehyde in 0.1% mol/l phosphate buffer, followed by 1% O_S_O_4_ overnight, dehydrated with a series of acetone, embedded and solidified. Then, the samples were sliced into 50-nm ultrathin sections to observe the intracellular structures via TEM HT7700 (Hitachi, Japan).

### RNA-fluorescence in situ hybridization (RNA-FISH)

The FISH assay was performed according to the manufacturer’s protocol (Genepharma, China). Briefly, the sections were reconstituted with a hybridization solution and hybridized with a digoxigenin-labeled KCNQ1OT1 probe (Genepharma, China) at 37 °C for 12 h. Then, the slides were incubated with a specific anti-DIG antibody (Boshide, China). Cell nuclei were counterstained with DAPI. The location and expression of KCNQ1OT1 were independently evaluated by two pathologists as: negative (−), positive (+ to ++).

### Luciferase reporter assay

The predicted miR-372-3p-binding sequences in KCNQ1OT1, *ATG5* 3′-UTR, and *ATG12* 3′-UTR was amplified by PCR and inserted into pmirGLO luciferase reporter vector (Promega, USA) to construct luciferase reporter vector, named as pmirGLO-KCNQ1OT1-wt, pmirGLO-*ATG5*-wt, and pmirGLO-*ATG12*-wt, respectively. Similarly, the potential binding sites of miR-372-3p in the above sequences were mutated by Quikchange Mutagenesis Kit (Agilent Technologies, USA) to construct mutant vectors, labeled as pmirGLO-KCNQ1OT1-mut, pmirGLO-*ATG5*-mut, and pmirGLO-*ATG12*-mut. H293T cells were seeded into 12-well plates at a density of 1 × 10^5^ cells/well. Twenty-four hours later, miR-372-3p mimics or scrambles were cotransfected with recombinant wide-type or mutant vectors by Lipofectamine 3000. The empty pmirGLO vector was transfected as control. The luciferase activities were standardized to the value of the cotransfected group with an empty vector and scrambles.

### Xenografts in nude mice

All animal experiments were approved by the Institutional Animal Experiment Committee at 920th Hospital. Four- to six-week-old nude mice were divided into different groups randomly (three mice/group). Then, 5 × 10^6^ cells, as indicated, were injected subcutaneously into the right forelegs of the mice. When a mean diameter of xenografts reached 6–8 mm, the mice were irradiated with a total dose of 12 Gy in two fractions during a course of 48 h. The tumor volumes were calculated every three days using the formula: *Volume* = (width^2^ × length)/2. The mice were killed four weeks after irradiation, and then the tumor masses were weighed. Efforts were made to ensure the animals suffered minimally.

### Histology and immunohistochemistry assays

The subcutaneous xenografts were fixed in 10% formalin, dehydrated with a series of acetone, embedded in paraffin. The samples from each treatment group were sliced into 4-μm slices and analyzed by hematoxylin and eosin staining. Immunohistochemistry (IHC) for Ki67 expression (anti-Ki67, 1:200, ab15580, Abcam) was performed as described previously^45^, and average Ki67 proliferative index of each group was determined by the ratio of the number of positive cells to the number of tumor cells in three random microscopic fields.

### Statistical analysis

The data were presented as means ± standard deviation (SD.) of three independent experiments and were analyzed statistically via SPSS 19.0 software. The differences between the two experimental groups were measured by two-tailed student’s *t*-tests, whereas ANOVA calculated differences among multiple groups. Pearson’s correlation test evaluated the linear relationship of gene expression. The relationship between gene expression and clinicopathologic parameters was analyzed by Chi-square test. *P* value <0.05 was considered to be statistically significant.

## Supplementary information

PCR Primers used in this study

KCNQ1OT1 was the most differentially expressed among the top 10 upregulated lncRNAs in IR-resistant cells
