# Supplementary material for: Upregulation of KCNQ1OT1 promotes resistance to stereotactic body radiotherapy in lung adenocarcinoma by inducing ATG5/ATG12-mediated autophagy via miR-372-3p
Source: Cell Death Dis. 2020 Oct 20;11(10):883. doi: 10.1038/s41419-020-03083-8 (PMC7575601; doi:10.1038/s41419-020-03083-8)
Supplement: Supplementary file 1 — PCR Primers used in this study [file 41419_2020_3083_MOESM1_ESM.docx]

**Table S1. PCR Primers used in this study**

| **Gene symbol** | **Sequence (5’-3’)** |
| --- | --- |
| KCNQ1OT1 | Forward: 5’-TACTGCCTTTCCTTCCGGTG-3’ |
|  | Reverse: 5’-CTGGTGTGACTGGAACCCTG-3’ |
| ATG5 | Forward: 5’-AGTTTTGGGCCATCAATCGG-3’ |
|  | Reverse: 5’-CAGCCACAGGACGAAACAGC-3’ |
| ATG12 | Forward: 5’-GGAGGGGAAGGACTTACGGA-3’ |
|  | Reverse: 5’-CAGCAGGTTCCTCTGTTCCC-3’ |
| GAPDH | Forward: 5’-CAGGAGGCATTGCTGATGAT -3’ |
|  | Reverse: 5’-GAAGGCTGGGGCTCATTT-3’ |
| miR-372-3p | Forward: 5’-AAAGTGCTGCGACATTTGAGCG-3’ |
|  | Reverse: 5’-AGTGCGTGTCGTGGAGTCG-3’ |
| miR-512-3p | Forward: 5’-CAAGTGCTGTCATAGCTGAGGTC-3’ |
|  | Reverse: 5’-AGTGCGTGTCGTGGAGTCG-3’ |
| miR-133b | Forward: 5’-TTTGGTCCCCTTCAACCAGCT-3’ |
|  | Reverse: 5’-AGTGCGTGTCGTGGAGTCG-3’ |
| miR-3605-5p | Forward: 5’-TGAGGATGGATAGCAAGGAAGCC-3’ |
|  | Reverse: 5’-AGTGCGTGTCGTGGAGTCG-3’ |
| miR-133a-3p | Forward: 5’-TTTGGTCCCCTTCAACCAGCTG-3’ |
|  | Reverse: 5’-AGTGCGTGTCGTGGAGTCG-3’ |
| U6 | Forward: 5’-CTCGCTTCGGCAGCACA-3’ |
|  | Reverse: 5’-AACGCTTCACGAATTTGCGT-3’ |
