# Supplementary figures and images for: Upregulation of KCNQ1OT1 promotes resistance to stereotactic body radiotherapy in lung adenocarcinoma by inducing ATG5/ATG12-mediated autophagy via miR-372-3p
Source: Cell Death Dis. 2020 Oct 20;11(10):883. doi: 10.1038/s41419-020-03083-8 (PMC7575601; doi:10.1038/s41419-020-03083-8)

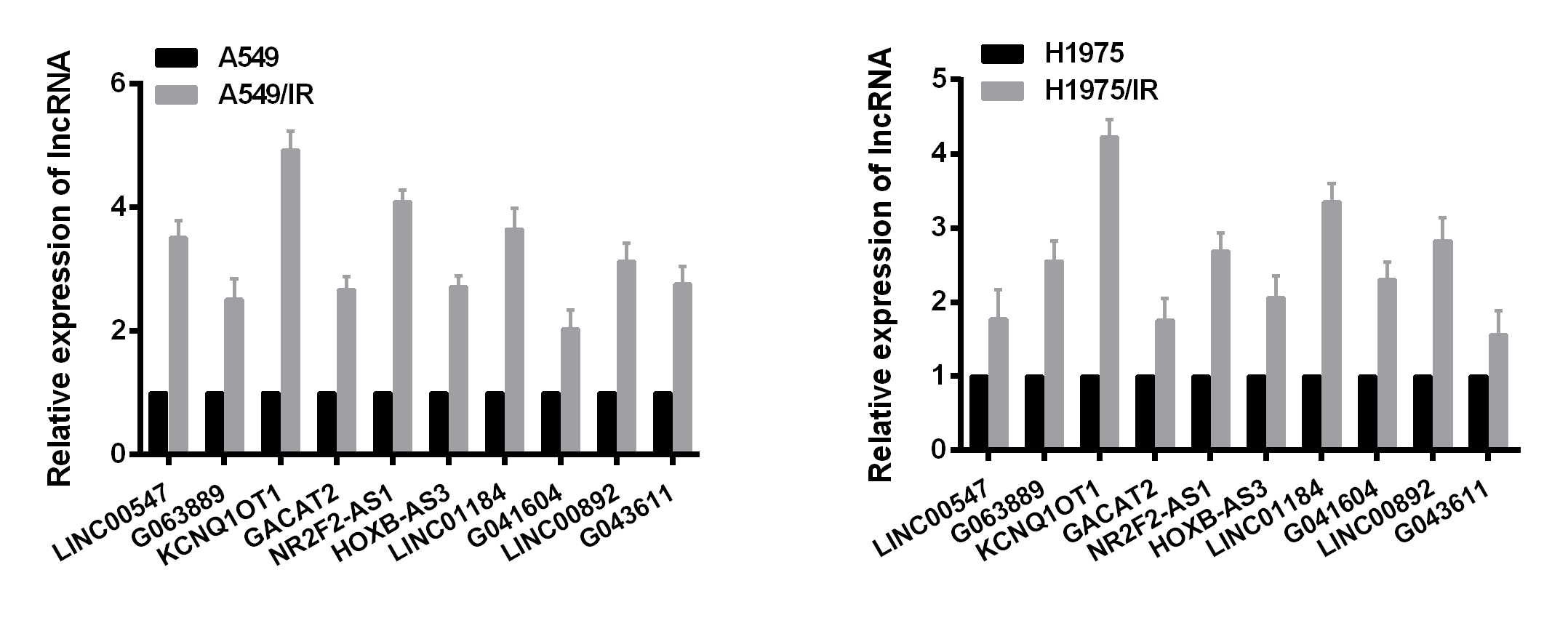

Supplement: Supplementary file 2 — KCNQ1OT1 was the most differentially expressed among the top 10 upregulated lncRNAs in IR-resistant cells [file 41419_2020_3083_MOESM2_ESM.tif]
